# Accuracy-Energy Configurable Sensor Processor and IoT Device for Long-Term Activity Monitoring in Rare-Event Sensing Applications

**DOI:** 10.1155/2014/546563

**Published:** 2014-12-14

**Authors:** Daejin Park, Jeonghun Cho

**Affiliations:** School of Electronics Engineering, Kyungpook National University, Daegu 702-701, Republic of Korea

## Abstract

A specially designed sensor processor used as a main processor in IoT (internet-of-thing) device for the rare-event sensing applications is proposed. The IoT device including the proposed sensor processor performs the event-driven sensor data processing based on an accuracy-energy configurable event-quantization in architectural level. The received sensor signal is converted into a sequence of atomic events, which is extracted by the signal-to-atomic-event generator (AEG). Using an event signal processing unit (EPU) as an accelerator, the extracted atomic events are analyzed to build the final event. Instead of the sampled raw data transmission via internet, the proposed method delays the communication with a host system until a semantic pattern of the signal is identified as a final event. The proposed processor is implemented on a single chip, which is tightly coupled in bus connection level with a microcontroller using a 0.18 *μ*m CMOS embedded-flash process. For experimental results, we evaluated the proposed sensor processor by using an IR- (infrared radio-) based signal reflection and sensor signal acquisition system. We successfully demonstrated that the expected power consumption is in the range of 20% to 50% compared to the result of the basement in case of allowing 10% accuracy error.

## 1. Introduction

In recent years, rare-event sensing systems [1] have been used in IoT-driven applications [2]. These systems feature internet connectivity, low-cost, very slow event-to-event duration, and long-lasting requirements. The IoT devices are used for activity monitoring [3], human sense interface [4], security monitoring, and medical applications [5]. The requirement for an extremely long lifetime is a critical issue in battery-operated IoT devices with a wireless connectivity.

The general-purpose microcontroller (MCU) has been widely used as a main processor of IoT-driven systems for sensor signal acquisition and data processing, which are not especially suited for rare-event sensing applications. The inefficiency of conventional MCUs in the sensing applications has been introduced in various literature [1, 6, 7] that reveal the key requirements of sensing application-specific architecture and processing methods. The latest studies of processor design for sensing applications are primarily based on architectural approaches that consider an event-driven nature [8] in extracting informative features from raw sensory data by observing over long periods of time.

**Figure 1:**
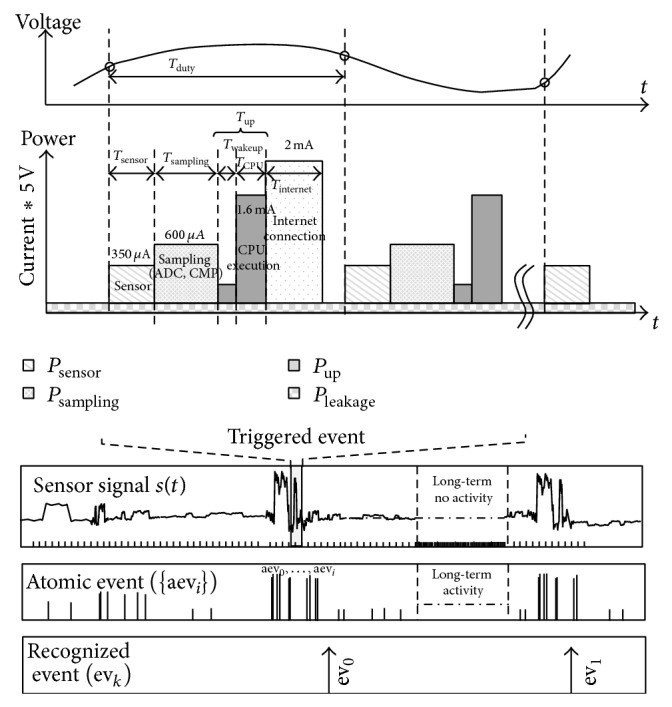
Power consumption for sensor signal sampling and processing in long-term activity monitoring.

Figure 1 illustrates basic operation and power consumption in a main sensor processor of the IoT device. A timely sampling operation to measure the transition of the target environment begins with activation of the sensor. An analog-to-digital converter (ADC) and level comparators perform analog data conversion, which generates digitized sample data and an interrupt request to execute the user-defined subroutines for digital signal processing. The CPU in the MCU is woken up to execute user-programmed interrupt service routines (ISRs), which are software code to analyze the quantized sensor data. Finally, the gathered data is transferred via internet connection to host systems.

The general-purpose MCU-based IoT device consumes inefficiently operating power in an iterative manner. Discrete-time-based sampling and signal processing are executed iteratively during the entire period of sensor signal transition, even during the period of long-term sleep. Although this weakness in terms of the power consumption can be resolved using several programming techniques where the interrupt handlers consider the sensor signal behavior, this is not formal approach that covers the general cases of signal characteristics.

Several approaches [6, 9–11] regarding the behavior of sensing applications have been introduced in sensor-based application for low power operations, especially in activity sensor, sensor processor, and wireless sensor nodes. However, the use of the discrete-time-based operation is restricted because the most feasible devices rely on commercial off the shelf (COTS) MCUs attached with several sensors. These devices attempt to control the ratio of the sleep mode to the active mode by switching the operation mode to lower the power consumption according to being with or without the sensory data, which are continuously determined by the discrete-time-based monitoring of the signal.

## 2. Motivation

To address this limitation of the conventional digital system architecture by using the discrete-time-based sensor data processing method, we propose an event-driven system architecture that modifies traditional digital system design. We present a theoretical framework to implement an event-driven sensor processor for general rare-event sensing applications by analyzing the system operations.

### 2.1. Event-Space Signal Representation

**Figure 2:**
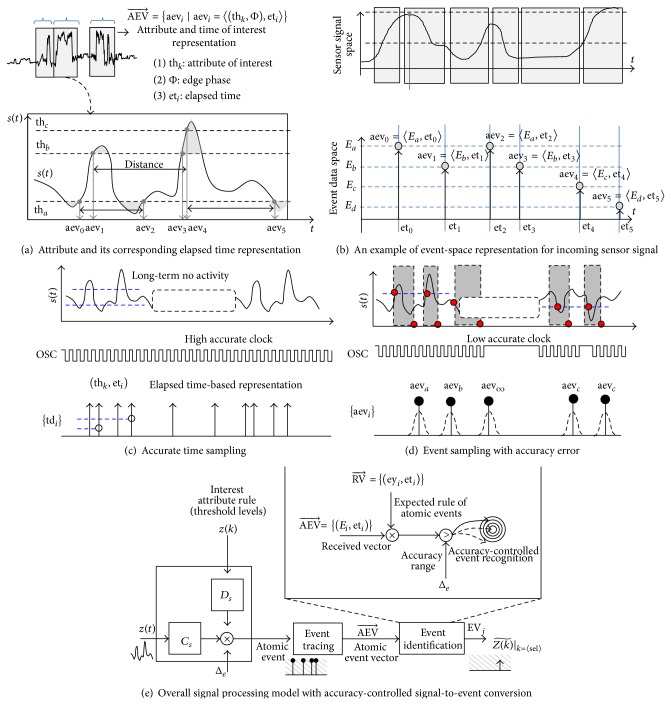
Event-space quantization and accuracy-configurable event-driven sensor processing flow.

Our main research begins with an event-space representation of the signal, instead of the digital data space domain.  Figure 2(a) illustrates fundamental signal elements using attributes of interest, and Figure 2(b) shows the event data space transition-based representation, which describes in detail the relationship between the featured points of the sensed signal.

The extracted features of the sensed signal are encoded into the elapsed time between events and informative value such as voltage level and edge phase crossing the trigger point of the signal. The fundamental event defined, which is defined as an atomic event with the most important information, provides a signal representation on an abstract level and reduces the computational complexity in performing basic data processing for extracted informative features of interest. The collected atomic events include partial information in the original signal that specifies whether the desired featured points of the signal are present.

### 2.2. Event-Quantization with Accuracy Error

The event-quantization concept extends the time-quantization method for signal representation that uses elapsed time to enhance the conventional data sampling and processing method. Time-quantization monitors only the specific conditions of the signal transition and captures the time-stamps. The event-quantization method also determines whether the specified characteristics of the signal exist. Figures 2(c) and 2(d) illustrate the difference between the time-sampling and event-quantization methods with an accuracy error, which monitors only the presence of the featured points of interest.

In terms of accuracy, there are two types of operations: timing accuracy and data resolution accuracy [12]. The former is dependent on the sampling frequency compared to the received signal bandwidth, and the latter is derived from the representative resolution of the sampled data. The timing resolution is dependent on the clock-duty resolution to resolve the timing window of the processing operations accurately.

Higher timing resolution for the duration measurement requires the accurate data processing in the time domain. However, in most cases, the data value resolution of the target system is tightly required, but time—related specification including the response time is relatively allowable in a certain range of error. In particular, in rare-event sensing applications, we assume that the timing measurement accuracy for the elapsed time between the arrived signal events can be performed with inaccurate operating clock relatively.

Although the events contain inaccurate time information, the final event receiver (for example, a human interface) is unable to identify the error differences compared to the ideal data within a certain error range [13].

### 2.3. Accuracy Configurable Signal-to-Event Conversion

The event-based approach, with a certain amount of accuracy error, is described by the proposed event-driven sensor data processing flow, as shown in Figure 2(e). The input signal is monitored with specified interest-of-signal characteristics *W*(*k*) to generate the specific atomic events aev_*i*_ of the signal. The set of atomic events during the specified region of the signal are traced into the tracer memory as an event vector $\overset{⃗}{\text{AEV}}$, which contains the sequence of the atomic events and the time distance relationship between the atomic events. The traced event vector identifies the approximate result $\tilde{Z(k)}$ as a final event by comparing it to the expected rules of the atomic events.

These approximation approaches enable us to reduce the computational complexity in order to manipulate a large amount of collected sensing data. As a result, power consumption will be reduced. For applications related to human interaction, an approximation approach enables developers to design the computational block using smaller hardware resources, while providing sufficient performance in limited resolution of the accuracy.

In accuracy-controlling approaches defined from the specifications, our study focused on the data-representation resolution, the timing resolution of the sampling frequency, and the response time as a delay time [14]. This enables the configuration of the operation accuracy in the processor architecture level according to the abstraction level of the proposed event-quantization approach.

## 3. Related Work and Constraints

To overcome the weakness of the frequent CPU wake-up in the discrete-time sampling, continuous time signal processing techniques [15, 16] have been proposed. If a certain condition of the signal status, such as the voltage level at a specific time, is matched with the user-defined condition [17, 18], the time value at the triggered condition is sampled and quantized [19] by the selective method, which also helps to reduce operational power [20].

**Figure 3:**
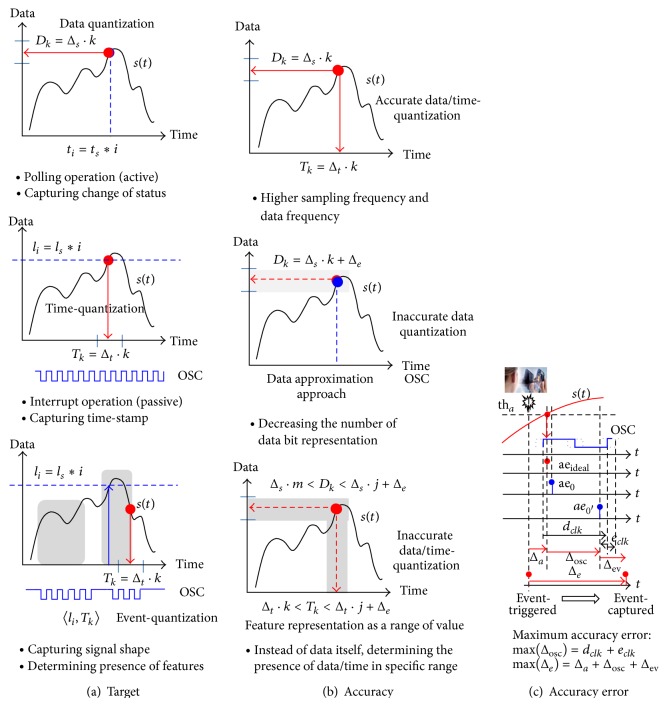
Category according to sampled target and its accuracy.

The continuous time sampling method illustrated in the second graph of Figure 3(a) requires additional hardware resources, including a dedicated oscillator and high-accurate timer block, to measure the elapsed time value instead of data sampling in high resolution. Event-driven signal processing based on the time-quantization method requires hardware overhead and more computational time for the time-distance calculation, which gives rise to additional power consumption. The required power and hardware resource overhead, which are needed to compensate for reduced wake-up power consumption, must be considered in order to achieve benefits in total energy efficiency due to hardware-energy trade-off.

The hybrid method, which uses a level triggered system wake-up and continuous time sampling scheme, monitors important signal transitions and performs detailed analysis using the discrete-time sampling method, which involves digital signal processing for second detail signal analysis. The proposed event-driven sensor data processing method tries to capture the signal shape instead of the elapsed time-stamp at the triggered point, as illustrated in the third graph of Figure 3(a).

The trade-offs in terms of energy and accuracy have been studied widely [21, 22]. To obtain long lifetime operations under limited battery power [23], the latest research introduces inaccurate computation techniques [12, 24] with approximation-based hardware designs, as described in the second graph of Figure 3(b).

The proposed sensing processor for the rare-event sensing applications adopts the event-driven approach of the continuous time-based sampling method. Inaccurate time-data manipulation, as shown in the third graph of Figure 3(b), reduces computational complexity and sampling resolution by determining the presence of featured events in the specific range. The level that allows an accuracy error in the time-stamp measurement is depicted in Figure 3(c). The level can be adjusted by making the trade-off between the processing energy consumption and the operating specification.

**Figure 4:**
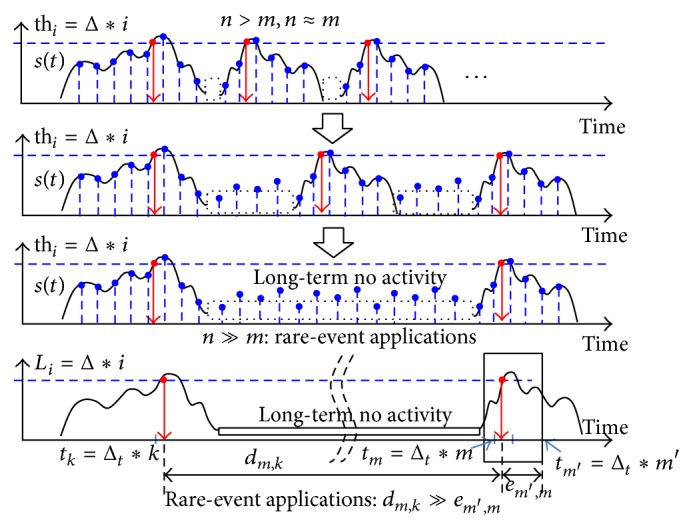
Wake-up frequency for data sampling and event-driven sampling.

Figure 4, shows the difference between the discrete-time samples and the featured events of interest, with the common shape of the rare-event sensor signal. Event sources, such as hand gesture, proximity, and object activity, generate signal pulses for which the distance between featured points of the signal is very long. The number of data samples (*n*) is greater than the number of events (*m*). In this work, we assumed an application-specific constraint of rare-event characteristics, which result in a small number of events compared to the number of data samples.

The event-quantization accuracy depending on the resolution of the elapsed time-stamp is described as *e*_*m*′,*m*_ in Figure 4. The rare-event sensing applications for which the event-to-event duration is relatively larger than the accuracy error have the following applications-specific constraints:

(1) $$\begin{array}{c} d_{m,k}\gg e_{m^{\prime },m}. \end{array}$$

With these application-specific constraints, the event identification accuracy error caused by the inaccurate time-stamp measurement clock is relatively insensitive derived from (1). The recognized event observer, such as human eye, allows a certain amount of inaccuracy in identifying a meaning of the events, which are constructed by the proposed inaccurate event-driven sensor processor.

The proposed sensor processor is designed with these application-specific constraints by reducing the accuracy of the time-stamp measurement clock, decreasing the bit width of the timer block to capture the time-stamps, and decreasing the operational complexity of the time-to-time distance measurement blocks, which are specially implemented as a dedicated accelerator for event recognition in the implemented hardware.

## 4. Proposed Architecture

### 4.1. Data-Time Sampling with Accuracy Error

**Figure 5:**
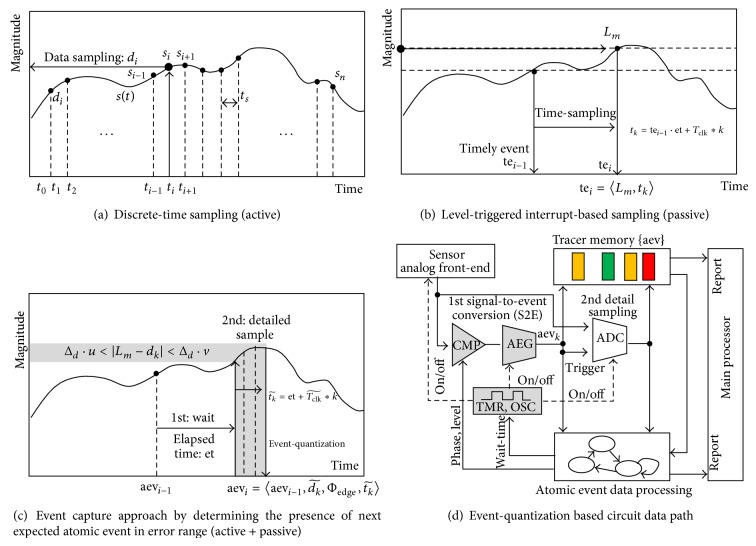
Event sampling illustrated and its circuit data path.

The first stage of the sensor processor is a sampler that gathers the time-variant information from the received signal. The conventional sampling method in Figure 5(a) attempts to collect all information in discrete-time from the target signal. There is no need to hold the time-stamp data. The uniformly sampled set in the time domain is described in the following definition.

**Definition 1 .**

Given continuous signal *s*(*t*), let *t*_*s*_ be a fixed sampling time and *S*_unitime_ = {*s*_1_, *s*_2_,…, *s*_*n*_} a uniformly sampled set in time domain *T* = {*t*_1_, *t*_2_,…, *t*_*n*_}. A sampled data *s*_*i*_ ∈ *S* and its data quantization result with error Δ_*d*_ by data quantization function DQ are defined as follows:

(2) $$\begin{array}{c} d_{i}\pm \Delta_{d}=\text{DQ}\left(s_{i}=s\left(t_{s}*i\right)\right). \end{array}$$

From this, the sampling time *t*_*s*_ in turn is defined as follows:

(3) $$\begin{array}{c} t_{s}=t_{i+1}-t_{i}. \end{array}$$

The quality of the data sampling toward zero Δ_*d*_ is dependent on the accuracy of the function DQ, which is usually implemented with ADCs or a comparator, and the resolution of sampling time *t*_*s*_.

The level-triggered interrupt-based sampling, as shown in Figure 5(b), tries to capture the time-stamp when it crosses a predefined condition with the parameterized value (such as the voltage level) and its transition edge phase.

**Definition 2 .**

Given continuous signal *s*(*t*), let *L* = {*L*_1_, *L*_2_,…, *L*_*n*_} be a set of all levels of interest to monitor data from *s*(*t*), TE a set of all pairs of the triggered level type and its elapsed time, TE = {te_*i*_∣te_*i*_ = 〈type, et〉,  *i* = 1,2,…, *n*}, and *T*_*clk*_ a fixed minimum period of the timer to measure the time-stamp at a triggered point. For the event te_*i*_ ∈ TE, sampled time *t*_*k*_ is elapsed time after the previous event te_*i*−1_ occurs, and its quantization result error Δ_*t*_ by the time-quantization function TQ is defined as follows:

(4) tk±Δt=TQtei·et=tei−1·et+Tclk∗k,1≤k<∞.

The elapsed time for the *i*th triggered event is recursively determined by searching the meet condition “*k*” of the following equation:

(5) tei·et=tei−1·et+tk,∀DQLm=DQstk, Lm∈L.

The time-stamp te_*i*_ · time resolution toward zero Δ_*t*_ is dependent on the minimum value of the time advance (*T*_*clk*_) and the number-of-bits representation of (*k*) value to encode the time value. Higher resolution of the time value requires continuous operations of the oscillator and timer unit with higher accuracy and large size of a timer counter unit to measure the time-stamp, which leads to energy consumption overhead as a side effect.

Figure 5(c) describes our approach to capture the signal shape as an atomic event crossing a certain range of arrival time. To more formally define our approach, we begin our explanation by first presenting the following definitions.

**Definition 3 .**

Given continuous signal *s*(*t*), let AEV = {aev_*i*_∣aev_*i*_ = (aev_*i*−1_, value, phase, et)} be a sequence of an atomic event aev_*i*_ crossing the specific level and time condition with a relationship of previous atomic event aev_*i*−1_, where aev_*i*_ · value is a result of the approximation-based data quantization function ADQ and aev_*i*_ · et is a result of the approximation-based time-quantization function ATQ, described as follows:

(6) dk~=ADQstk,Lm,Δd,u,v,∀Δd∗u<Lm−dk<Δd∗v,tk~=ATQaevi·et,Tclk~,where  Tclk~=Tclk+Δt.

The meet condition *k*, when the expected crossing is present, is described in the following equation:

(7) $$\begin{array}{c} \tilde{t_{k}}=\text{et}+\tilde{T_{clk}}*k,\;\forall \text{DQ}\left(s\left(\tilde{t_{k}}\right)\right)=\tilde{d_{k}}. \end{array}$$

The atomic event generator (AEG) builds an element with the attributes, which are encoded with the digitized signal level, elapsed time, and edge phase in the following equation:

(8) $$\begin{array}{c} \text{AEG}\left(s\left(t\right),L\right)=\left\{\text{ae}{\text{v}}_{i}\;|\;\text{ae}{\text{v}}_{i}=\langle \text{ae}{\text{v}}_{i-1},\tilde{d_{k}},\phi_{\text{edge}},\tilde{t_{k}}\rangle \right\}. \end{array}$$

From (8), the extracted information, as an atomic event, is encoded with the approximation value of the signal level, the reduced time-quantization value of the elapsed time, and the relationship of the previous atomic event aev_*k*−1_.

Figure 5(d) shows the proposed hardware data path for the atomic-event sampling in Figure 5(c), including the level comparators, timer, oscillator, and atomic event generator (AEG) implementing operations from (2) to (8).

### 4.2. Atomic Event Segmentation

**Figure 6:**
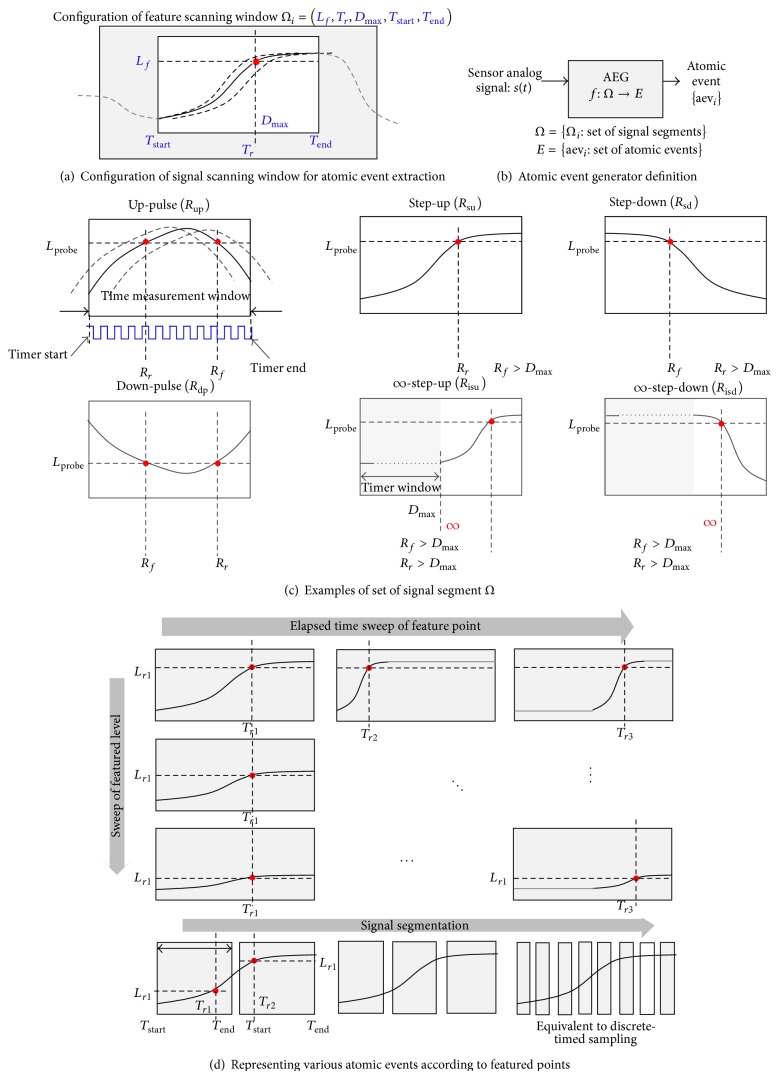
Configuration of signal feature scan window and atomic event segmentation.

Atomic event generation is a method to represent a certain range of the continuous signal pattern with an abstract event. The signal representation is classified by a user-defined set of signal segments. We provide an example in Figure 6. The feature scan window in Figure 6(a), which is used to capture the atomic event of the signal, is configured with a specific voltage level, time window, and elapsed time at the feature point. The configured scan window determines if the featured points are monitored in the snapshot of the signal passing through the configured scan window and generates an element of a set of atomic events in Figure 6(b).

**Definition 4 .**

Given the configured feature scanning window to extract the atomic events from *s*(*t*), let *T*_start_ be a start time monitoring the signal, let *T*_end_ be the end of monitoring the signal, let *L*_*r*_ be a rising signal level at which the time-stamp is *T*_*r*_, let *L*_*f*_ be a falling signal level at which the time-stamp is *T*_*f*_, let the pair of *L*_*x*_ and *T*_*y*_ be featured point, and let *D*_max⁡_ be a maximum time value in which the featured points are present. The set of signal segments described by the configuration *Ω* = {*Ω*_*i*_∣*Ω*_*i*_ = (*T*_start_, *T*_end_, *L*_*r*_, *L*_*f*_, *T*_*r*_, *T*_*f*_, *D*_max⁡_)} of the featured scanning window is defined as *Ω* and is used to extract the atomic events of interest for the AEG function, which is defined as follows:

(9) $$\begin{array}{c} \left\{\text{ae}{\text{v}}_{i}\right\}=\text{AEG}\left(s\left(t\right),\Omega \right). \end{array}$$

*Ω*_up_ defines a signal segment of the feature scanning window as an example showed in “Up-Pulse” shape of Figure 6(c). In our applications, {*Ω*_type_∣type  = “up ”, *“*su ”, *“sd*⁡ ”, *“*dp ”, *“*isu ”, *“*isd ”} is used.

Figure 6(c) shows examples of the user-defined signal shape as an atomic event, which is determined by the configured feature scan window. The “up-pulse” pattern rises at recognized time *R*_*r*_ and falls at recognized time *R*_*f*_ for the *L*_probe_ level during maximum timer window *D*_max⁡_. The continuous signal shape during a configured time range of *T*_start_ and *T*_end_ is represented as abstract event *R*_up_ with the following equation:

(10) $$\begin{array}{c} \text{AEV}\left(s\left(t\right),\Omega_{\text{up}}\right)≃\text{ae}{\text{v}}^{\text{up}}=\langle \Omega_{\text{up}},L_{\text{probe}},\left(R_{r},R_{f}\right)\rangle . \end{array}$$

The expected rule to identify aev^up^ atomic event pattern, allowing time error margin Δ, is represented by the following equation:

(11) $$\begin{array}{c} R^{\text{up}}=\left(\text{ae}{\text{v}}^{\text{up}},\Delta \right). \end{array}$$

The “step-up” pattern rises at time *R*_*r*_ and does not fall within the timer window *D*_max⁡_. The continuous “step-up” pattern signal can be also represented by the abstract atomic event view in the following equation:

(12) $$\begin{array}{c} \text{AEV}\left(s\left(t\right),\Omega_{\text{su}}\right)≃\text{ae}{\text{v}}^{\text{su}}=\langle \Omega_{\text{su}},L_{\text{probe}},\left(R_{r},-\right)\rangle . \end{array}$$

The *∞*-step-up pattern includes the specific range of no signal transition crossing the specified voltage level *L*_probe_ within maximum timer window *D*_max⁡_ and a rise at any time. The continuous *∞*-step-up pattern signal can be also represented by the abstract atomic event in the following equation:

(13) AEVst,Ωisu ≃aevisu=Ωisu,Lprobe,∞,∞,Rr.

The aev^isu^ atomic event pattern is also represented by the expected atomic event pattern rule and its time error margin using the following equation:

(14) $$\begin{array}{c} R^{\text{isu}}=\left(\text{ae}{\text{v}}^{\text{isu}},\Delta \right). \end{array}$$

The *∞*-step-up pattern is a powerful method to simplify the representation of the long-term signal shape with no activity, which leads to a reduction in the capacity of the information.

Figure 6(d) shows the capability to represent various signal shape by the configuration of *L*_*r*_, *D*_max⁡_, *T*_*r*_, *T*_start_, and *T*_end_ in the feature scan window. One signal shape can be divided into the several slices by user-defined signal segmentation. If the time window for signal segmentation is the same as the fixed width *t*_*s*_ in the discrete-time sample method, the result of the atomic event generation is equivalent to that of the discrete-timed sampling. The proposed atomic event generation approach enables a trade-off between the signal extraction accuracy and its processing power consumption.

### 4.3. Event-Driven Data Processing

**Figure 7:**
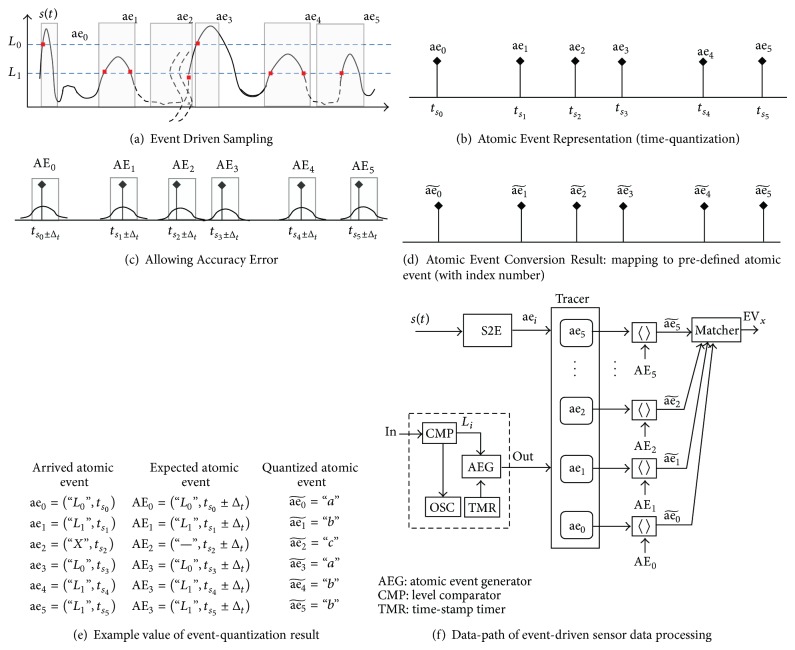
Event-driven sensor data processing concept for macro-level signal analysis.

The atomic event generator (AEG) scans the continuous signal *s*(*t*) passing through the configured feature scan window to determine the presence of the signal shapes of interest, as shown in Figure 7(a). The set of atomic events is generated with a pair of attributes and time-stamps as a result of the time-quantization shown in Figure 7(b). Consider

(15) $$\begin{array}{c} \text{aev}=\left\{\text{ae}{\text{v}}_{i}\;|\;\text{ae}{\text{v}}_{0},\text{ae}{\text{v}}_{1},\ldots ,\text{ae}{\text{v}}_{i}=\left(“L_{i}\;\text{”},ts_{i}\right)\right\}. \end{array}$$

Figure 7(c) shows a signal representation by a set of atomic events with a certain amount of error. This is denoted in the following equation:

(16) $$\begin{array}{c} \tilde{\text{ae}}=\left\{\tilde{\text{a}{\text{e}}_{i}}\;|\;\tilde{\text{a}{\text{e}}_{0}},\tilde{\text{a}{\text{e}}_{1}},\ldots ,\tilde{\text{a}{\text{e}}_{i}}=\left(“L_{i}\;\text{”},\bar{ts_{i}\pm \Delta }\right)\right\}. \end{array}$$

aev_*i*_, which is matched with the configured scan window AE_*i*_, is represented as an abstracted atomic event index in Figures 7(d) and 7(e), which indirectly address the detailed attributes in the constant dictionary. The continuous analog signal is converted into a set of event quantized data $\tilde{\text{ae}{\text{v}}_{i}}$, and its index value is traced only into the atomic event tracer buffer. Therefore, the traced event data processing manipulates the index value and its relationship to the representative atomic events to generate the final event EV. The proposed event-driven sensor data processing unit (EPU), which is based on event-quantization, provides the following advantages compared to conventional sensor data processing:

- representing the continuous analog signal with a small number of atomic events for specially featured points,
- decreasing the number of pieces of processing data with reduced atomic events,
- allowing the accuracy error of the generated atomic events for application-specific properties of the rare-event sensing applications,
- decreasing the complexity of the expected atomic event comparison circuit, by comparing the time-stamp range, instead of the accurate value,
- mapping the recognized atomic events into the representative atomic event set with only index value,
- transforming the raw data processing into the index value.

**Figure 8:**
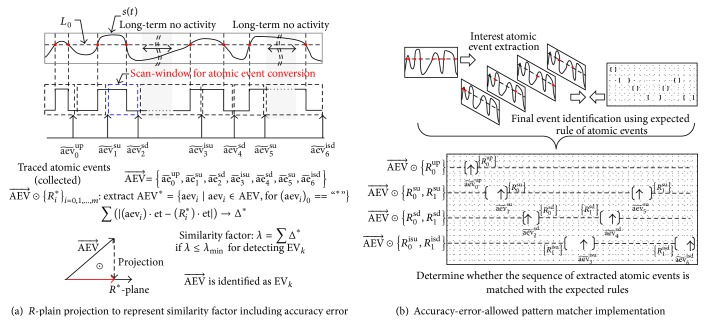
Event-print identification for atomic event of interest.

Figure 7(f) illustrates the corresponding data path of the event-driven sensor data processing, including the atomic event generator, tracer, feature scan window, and the pattern matcher (which is described as event-print window matcher in Figure 8(b)).

### 4.4. Final Event Identification

The archived atomic events in the tracer memory are evaluated as a similarity factor, which is calculated by the total sum of the distance between the collected events and the expected rule. We define this procedure as *R* ^*^-plain projection, as illustrated in Figure 8(a). The atomic event extraction procedure is described by the $\overset{⃗}{\text{AEV}}$ in the following equation. The operation ⨂ describes the atomic event conversion for the continuous sensor signal *s*(*t*) with the atomic event conversion rule, which is introduced in Figure 6(c). Consider

(17) $$\begin{array}{c} s\left(t\right)⨂L_{0}=\overset{\vec{}}{\text{AEV}}. \end{array}$$

In the signal example of Figure 8(a), the result of atomic event generation is described by the following equation:

(18) AEV⃗=aev~0up,aev~1su,aev~2sd⁡,aev~3isu,aev~4sd⁡,aev~5su,aev~6isd.

Figure 8(b) shows the proposed event-print window matcher implementing the *R* ^*^-plain projection to determine the final event using the result based on the similarity factor. The event-print matcher allows a certain error comparing the arrived atomic events to the expected rule, which is illustrated as blank holes in the punch card of Figure 8(b). Multiple *R* ^*^-plain projections are performed simultaneously to compare the archived atomic events with various pattern rules. We define these matching operations ⨀ with the following equation:

(19) $$\begin{array}{c} \overset{⃗}{\text{AEV}}⨀{\left\{R_{i}^{*}\right\}}_{i=0,1,\ldots ,m}. \end{array}$$

The extracted atomic event vector is described by the following equation:

(20) AEV∗→=aevi∗ ∣ aevi∈AEV, foraevi·type==“∗”.

The difference between the elapsed time stamp value and expected event arrival value is calculated for the extracted atomic events list using the following equation:

(21) $$\begin{array}{c} \Delta_{i}^{*}=\left|\left(\text{ae}{\text{v}}_{i}^{*}\right)\cdot \text{et}-\left(R_{i}^{*}\right)\cdot \text{et}\right|. \end{array}$$

The operation ⨀ of the extraction rule *R*_i_^*^ for atomic event vector $\overset{⃗}{\text{AEV}}$ can be considered as the *R*_i_^*^ plane projection.

The total similarity factor, which is compared to the expected event rules, is described as the summation of the difference in the measured time-stamp:

(22) $$\begin{array}{c} \lambda =\sum \Delta_{i}^{*}. \end{array}$$

If the final value *λ* is less than the minimum value of the expected error range, the received atomic event $\overset{⃗}{\text{AEV}}$ is identified as EV_*k*_. The trade-off between the processing accuracy and corresponding energy consumption can be selected to satisfy the design specification, which is described by the functional constraints and the required operating lifetime.

## 5. Implementation and Experimental Results

**Figure 9:**
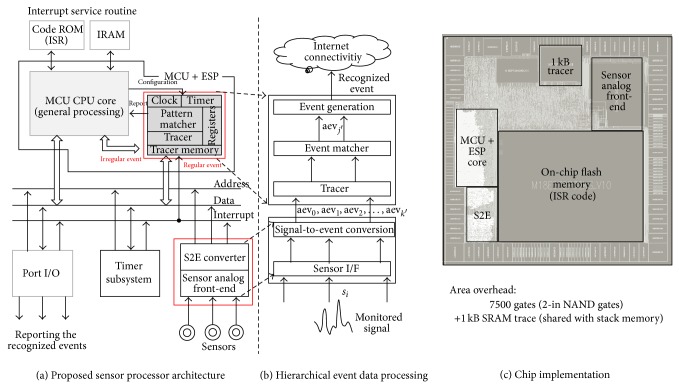
VLSI IC implementation of microcontrollers with the proposed event signal processing unit (EPU).

The proposed event-driven sensor data processing unit (EPU) is implemented as an accelerator to perform energy-efficient event recognition from the incoming sensor signal, as described in Figure 9(a). The regular case for sensing data analysis can be covered by the proposed event processor, which enables the MCU core to hibernate during sleep mode. The user-defined software configured by the MCU core allocates the configuration of the predefined atomic event conversion conditions for the feature scan window.

The set of atomic events is redirected into the tracer buffer via the dedicated DMA bus. Atomic event generation and event vector construction are performed in silent background mode, without waking up any of the main MCUs.

The newly designed event-driven sensor processor, including the general-purpose MCU core and the EPU core, is implemented using 0.18 *μ*m CMOS embedded-flash process technology and has a die size of 1.2 mm × 1.2 mm, as shown in Figure 9(b). The proposed method requires, approximately, an additional 7500 logic gates using a 2-input NAND for the timer counter, signal-to-event converter (S2E) including AEG blocks, event-print window matcher in EPU unit, and 1-KB SRAM buffer for the atomic event vector tracer.

**Figure 10:**
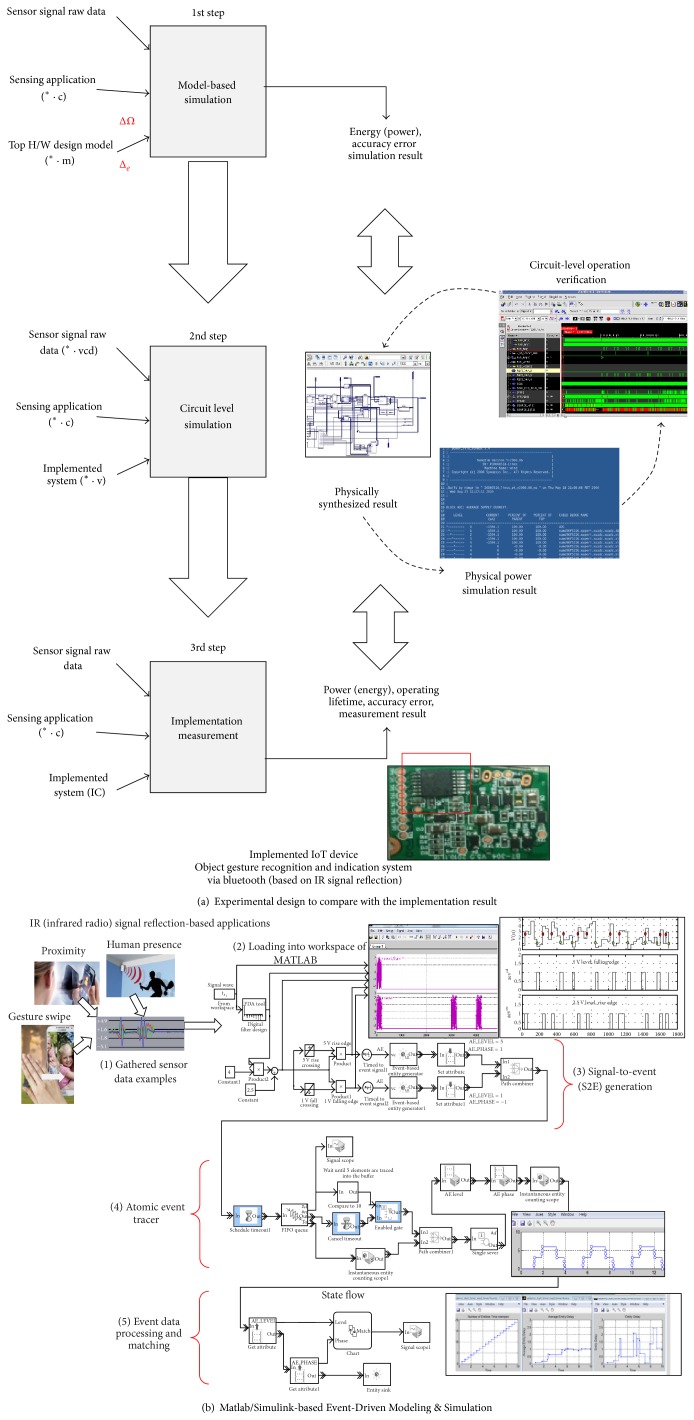
Experimental design and measurement of the implementation result.

Figure 10(a) shows the experimental design and measurement method to validate the efficiency in processing energy consumption by the proposed method and its implemented hardware. The first step of the evaluation is performed at the simulation level using the Matlab/Simulink models.

The physical sensor signal acquisition by the real activity (such as gesture swipe, proximity, and human presence) is performed off-line to save the raw dump file of time-variant sensor signal. Then the raw file of the archived sensor signal is loaded into the Matlab workspace.  Figure 10(b) shows that the proposed event processing flow is evaluated by the model-based designs using discrete-event design tool sets supported by the Simulink.

The second step of the evaluation is performed by the circuit-level simulation for the synthesizable hardware design. The proposed method and its hardware architecture are implemented with the fully synthesizable Verilog RTLs, which can be physically mapped onto the FPGA or CMOS silicon chip.

The implemented chip includes the test mode interface, which requires about 1800 logic gates, to access the on-chip registers and the bus transactions in supervisor mode. If the predefined test sequences are forced into the input ports on the power-up duration, the chip is entered into the supervisor mode in which the important nodes of the system can be accessed by the external interface. The user-driven external trigger events are loaded into the on-chip via the test mode interface to emulate the dynamic operation executing user applications. The event detection timing in chip-level is compared to the expected timing and the power consumption is also measured in the real environment by the user-triggered events. The hardware overhead for the test mode interface will be excluded in a final chip for the mass production.

The third step compares the simulation results with the electrical results, which are measured for the implemented IoT sensor device, including the proposed sensor processor. Because the gate-level synthesized design files are nearly equivalent to the physical hardware, the power simulation results show that the proposed sensor processor architecture may reduce energy consumption.

The implemented IoT sensor device is a type of IR- (infrared radio-) based standalone system that senses a change in the movement of an object. The IR transmitter, IR receiver, the proposed sensor processor, Bluetooth for the wireless connectivity, and on-board battery are integrated on a single tiny PCB board, as shown in the board screenshot of Figure 10(a).

The IR transmitter generates a specific pattern of signal pulses. The IR receiver acquires the light signal reflected by the target objects, and the implemented sensor processor performs signal processing to analyze the meaning of the signal.

**Figure 11:**
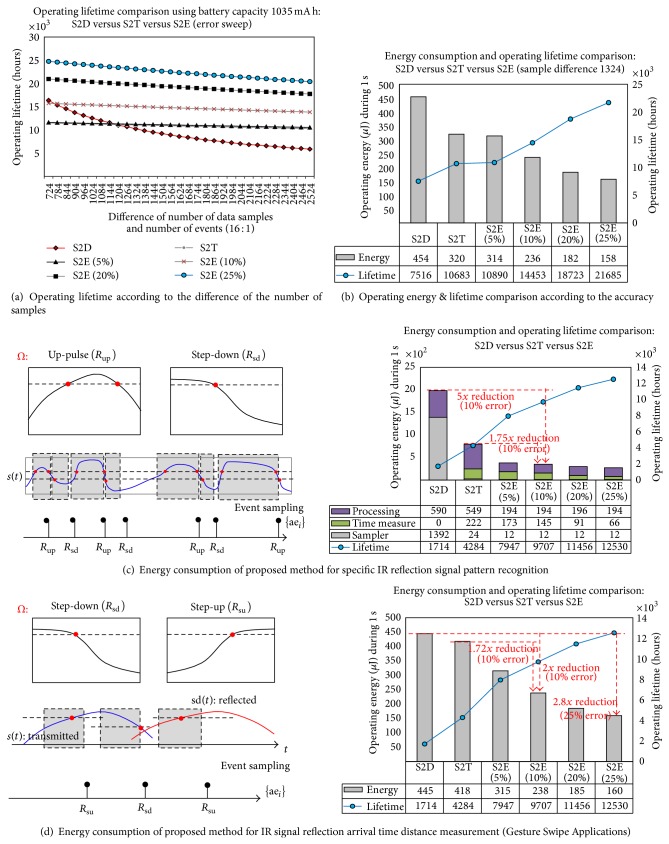
Experimental results.

Figure 11 shows the experimental results by the implemented IoT device, comparing the operating lifetime according to the number of sample differences by the processing method in Figure 11(a). The S2D describes the result by the conventional polling-based sensor data processing method. The S2T shows a result from the level-triggered interrupt-based signal processing method. The S2E shows the results from our proposed method. The improved results from allowing an accuracy error are also evaluated. These results, which allow for a 25% error variation in the specific constraints in our applications, are shown in Figures 11(a) and 11(b).

All building blocks in the implemented IoT sensor device hibernate during sleep mode except that of the EPU (including the signal-to-event converter), which is only active in order to trace the transition of the incoming signal by comparing the signal features of interest. When the final event is identified by the event-print window matcher, the main MCU wakes up to activate the subsequent building blocks to transfer the recognized events to a host system using the wireless connectivity.

As sensing applications of the implemented IoT sensor device, the low-power recognition performance based on the proposed method is evaluated in terms of its energy consumption.  Figure 11(c) shows the results for the specific IR sensor signal recognition using a defined set of signal segments *Ω*. The figure shows an 80% reduction when a 10% accuracy error is allowed, compared to the original work.

Figure 11(d) shows the energy-efficient recognition of the hand-swipe gestures, which are described by the two types of signal segments *Ω* and show a 50% reduction when a 10% accuracy error is allowed, compared to the original work. The reduction in energy consumption is achieved by configuring the implemented architectural framework with application-specific constraints that allow the required recognition accuracy error.

The margin of the acceptable accuracy error is dependent on the application-driven requirement. The trade-off between the event detection accuracy and low-power consumption has to be considered in implementing the IoT device. The proposed chip architecture provides the configuration register to allow the user-defined accuracy error for more long-term operation of the IoT device. In the environmental monitoring applications, such as proximity, hand gesture, temperature, and light intensity, the response error in less than several seconds for event detection is small enough to allow the 50% accuracy error.

## 6. Conclusion

The proposed event-driven sensor processor architecture for sensing applications with rare-event constraints is proposed and implemented as an accelerator. This enables the sensor signal processing in an energy-efficient mode by allowing for the accuracy error, which is caused by the abstraction of the original signal as atomic events. All of the building blocks, including atomic event generators and the EPU core, are implemented as a single system-on-a-chip, which is integrated into the IoT sensor device.

The proposed method uses the characteristics of rare-event sensing applications, in which timing accuracy error is relatively insensitive in sensor signal recognition and introduces the concept of atomic event generation as a method of event-quantization. The event-space representation of the sensor signal by the extracted set of atomic events is constructed with a user-defined event signal segmentation by the configured feature scan window.

The event-quantization result is archived as a set of unique indexes into the tracer buffer. The event-print matcher determines the presence of the featured signal points for the collected atomic event vector, in order to identify the event from the original sensor signal. The event-matching process is based on the similarity factor calculation, named *R* ^*^-plain projection that describes the expected rules of the signal patterns.

The implementation results, which are evaluated for an IR-based signal recognition system for object activity monitoring applications, show a reduction in total energy consumption by delaying the activation of the main processor and the Bluetooth interface for the wireless connectivity. The proposed sensor processor provides an architectural framework by providing an application-specific configuration of the event-quantization level for the energy-accuracy trade-off. This results in additional benefit of the energy consumption, which maximizes the operating lifetime of the IoT sensor device.
